# Supplementary material for: Genome-Wide Identification of the MAPK and MAPKK Gene Families in Response to Cold Stress in Prunus mume
Source: Int J Mol Sci. 2023 May 16;24(10):8829. doi: 10.3390/ijms24108829 (PMC10218611; doi:10.3390/ijms24108829)
Supplement: Supplementary file 1 [file ijms-24-08829-s001.zip › ijms-2209574-supplementary/Figures S1-S9.pdf]

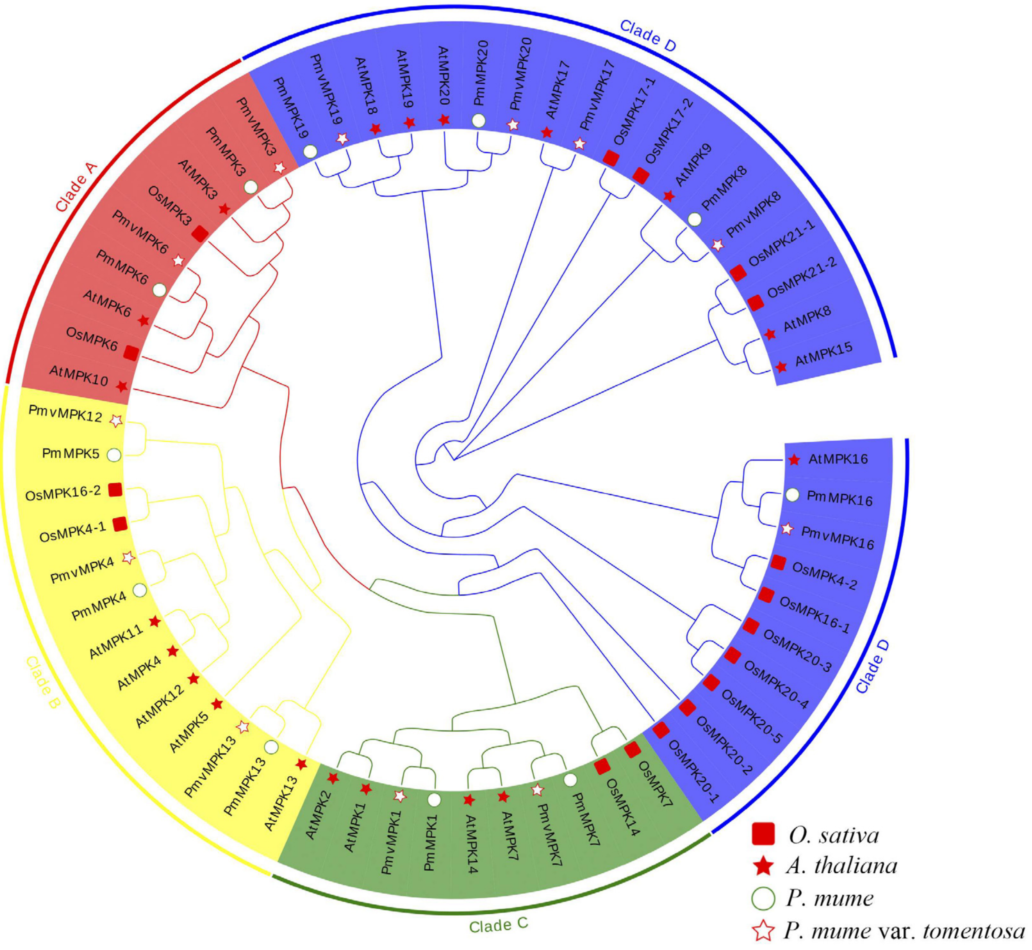

Figure S1 Phylogenetic trees of *AtMPKs*, *PmMPKs*, *PmvMPKs* and *OsMPKs*. Different species are indicated with shapes and colors.

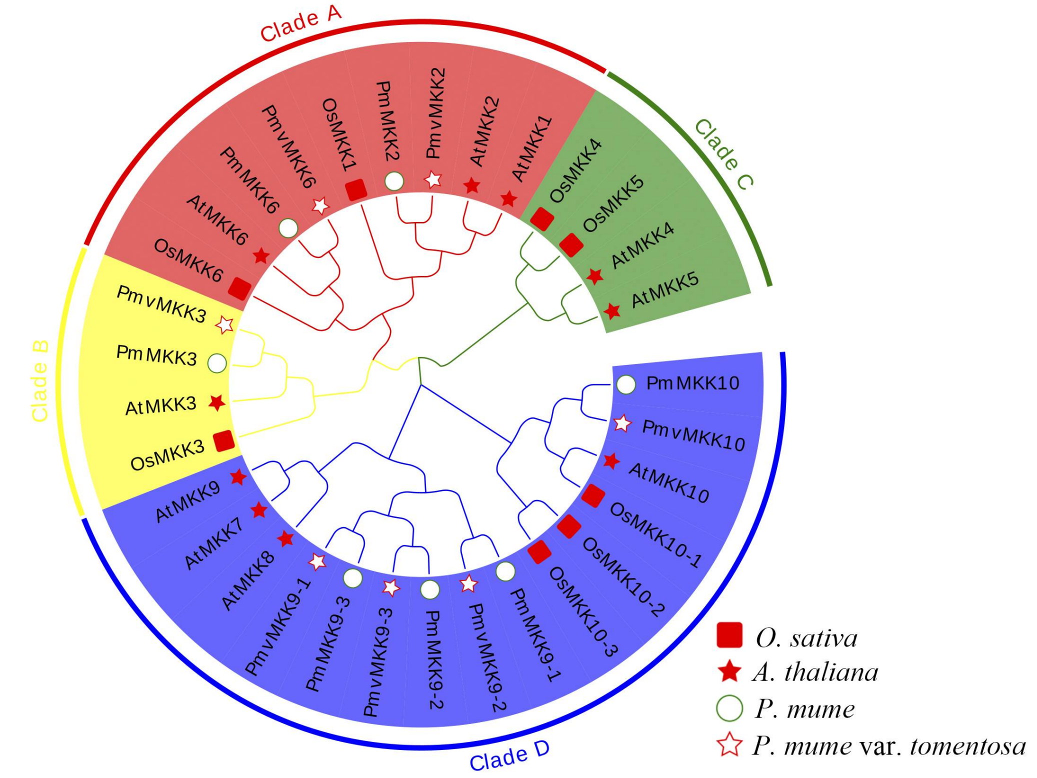

Figure S2 Phylogenetic trees of *AtMKKs*, *PmMKKs*, *PmvMKKs* and *OsMKKs.* Different species are indicated with shapes and colors.

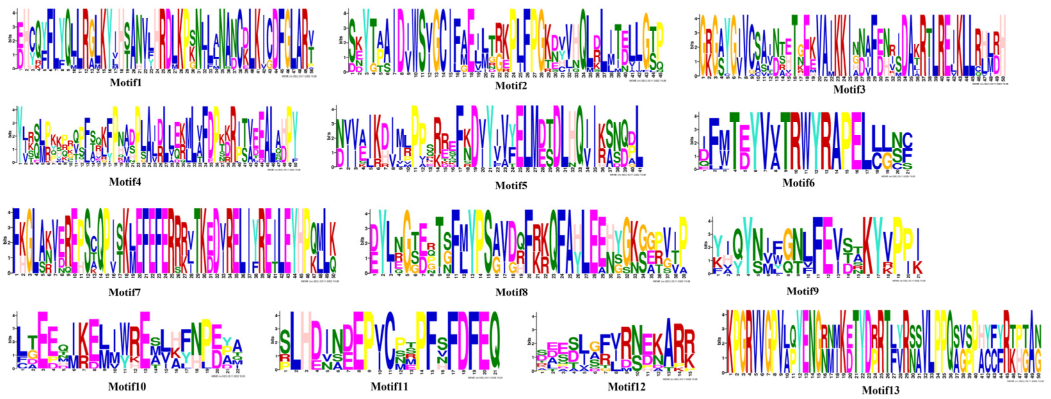

Figure S3 Schematic diagram of *Pm*MPK and *Pmv*MPK protein motifs

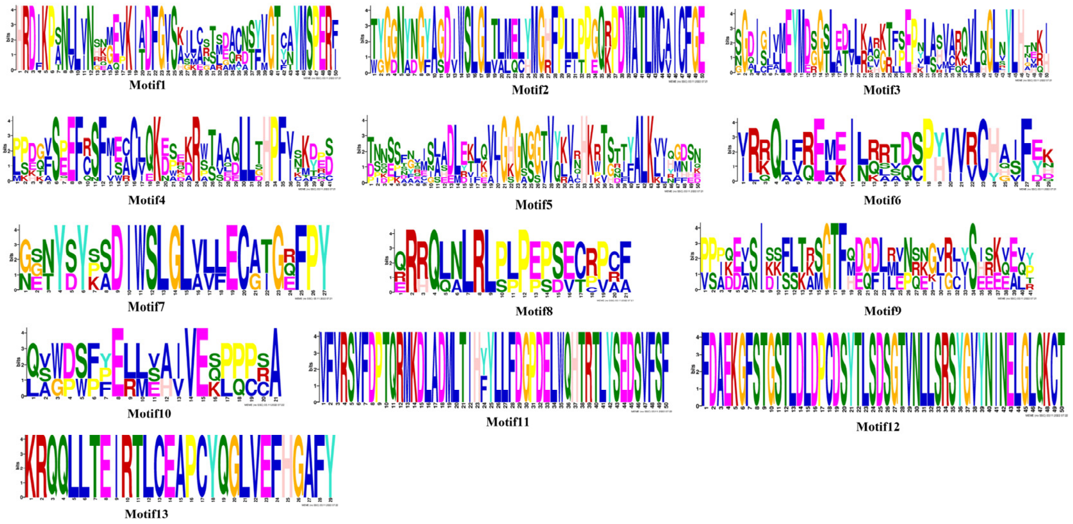

Figure S4 Schematic diagram of *Pm*MKK and *Pmv*MKK protein motifs

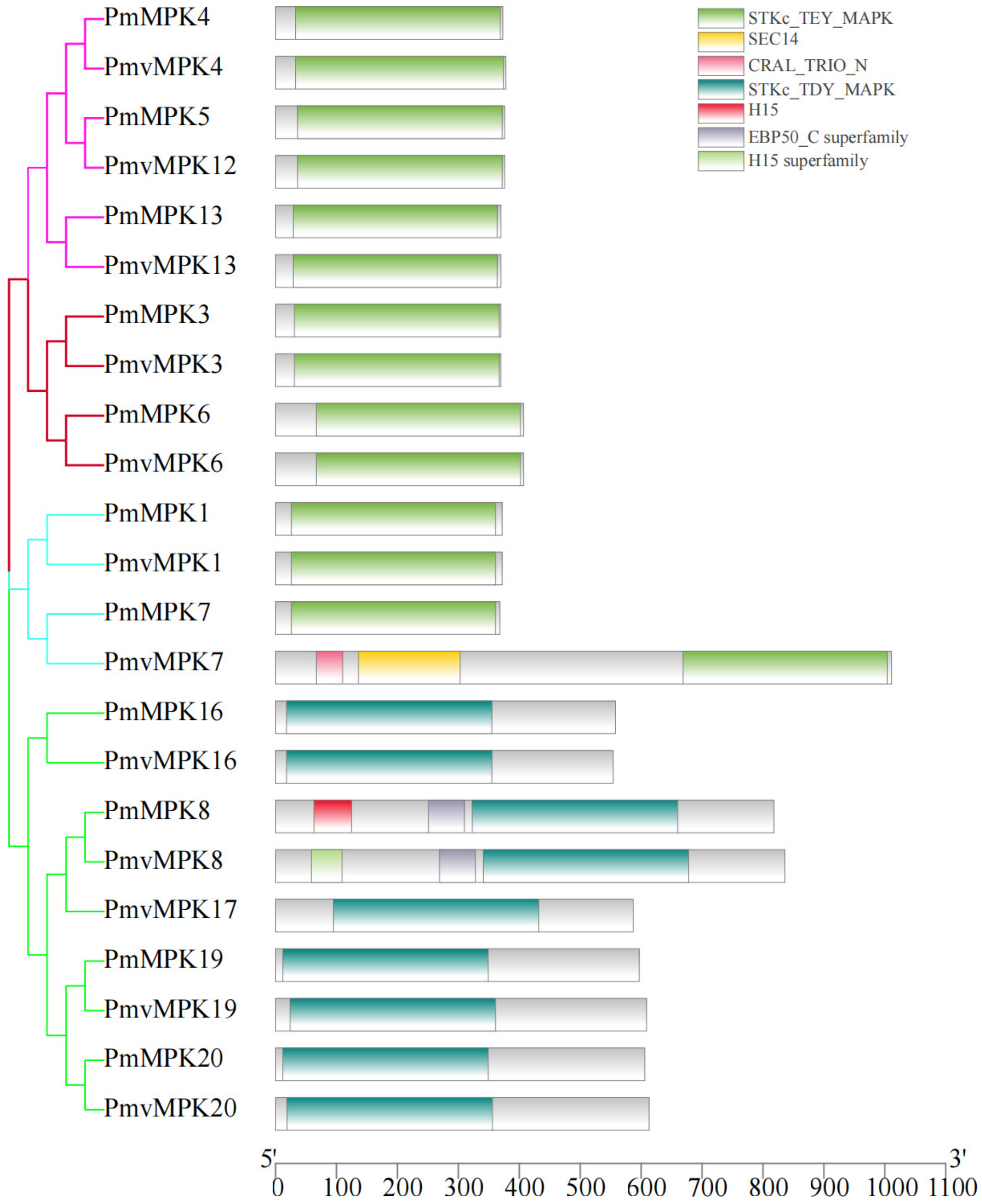

Figure S5 The conserved domains in *Pm*MPK and *Pmv*MPK proteins

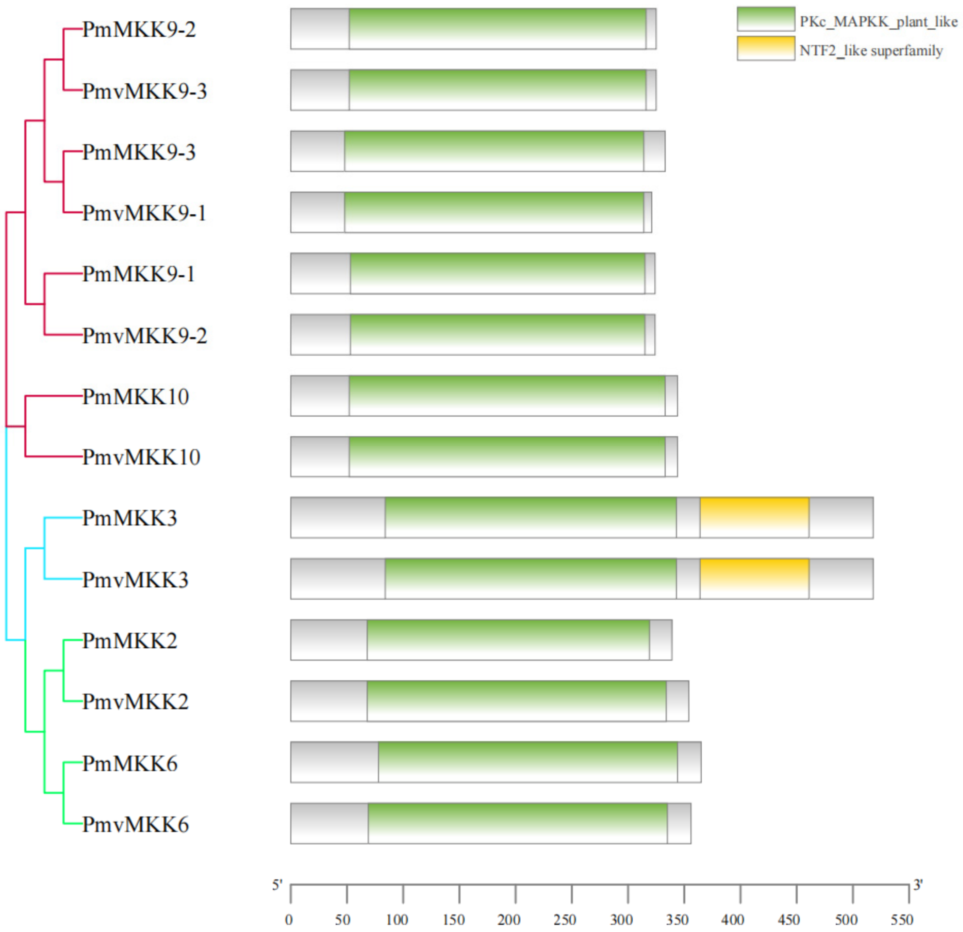

Figure S6 The conserved domains in *Pm*MKK and *Pmv*MKK proteins

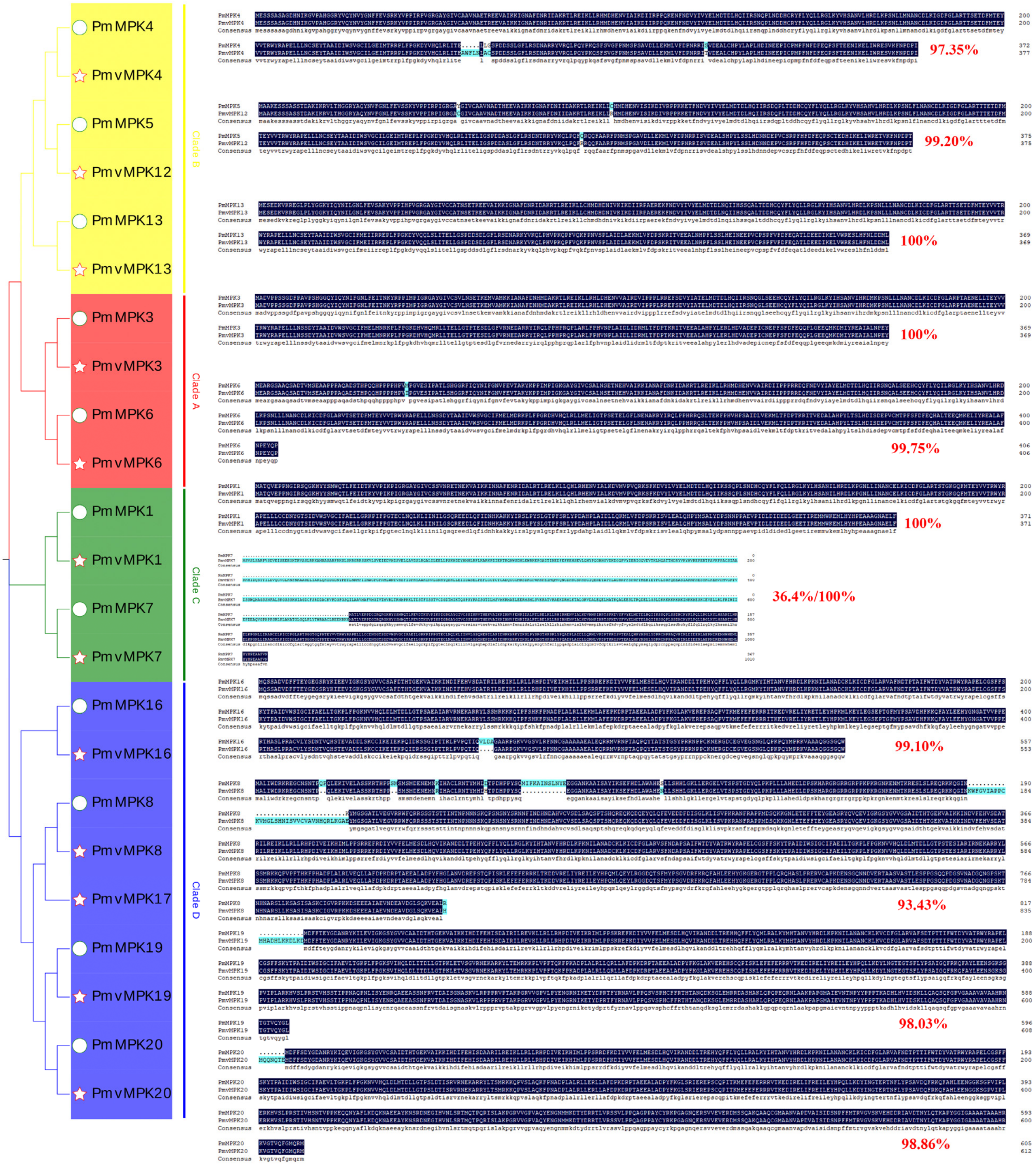

Figure S7 Sequence alignment and similarity ratio of *PmMPK* and *PmvMPK* homologous gene

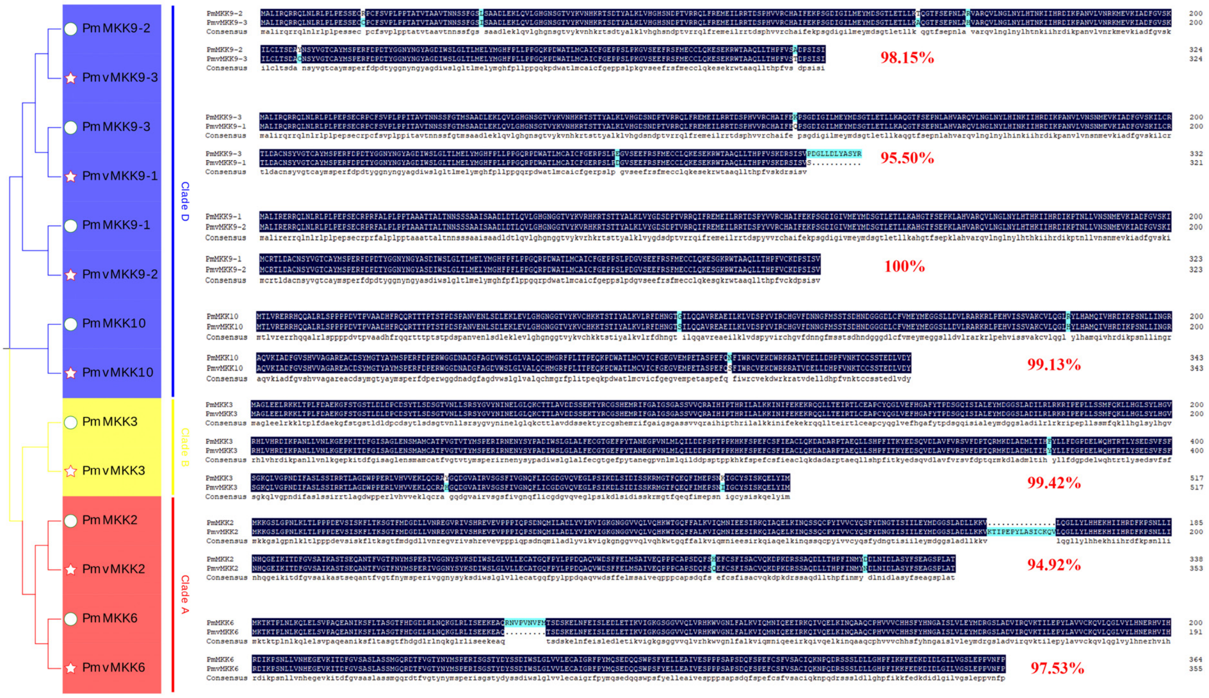

Figure S8 Sequence alignment and similarity ratio of *PmMKK* and *PmvMKK* homologous gene

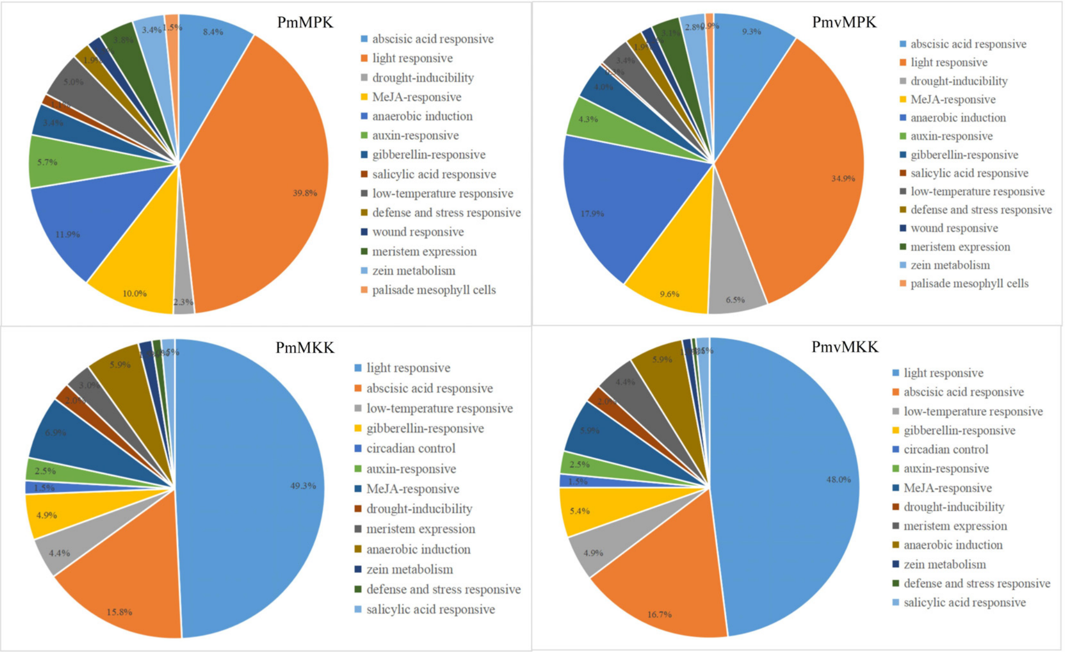

Figure S9 The proportion of all cis-elements predicted in the promoters of *MPKs* and *MKKs* in *P. mume* and *P. mume* var. tortuosa using PlantCARE website

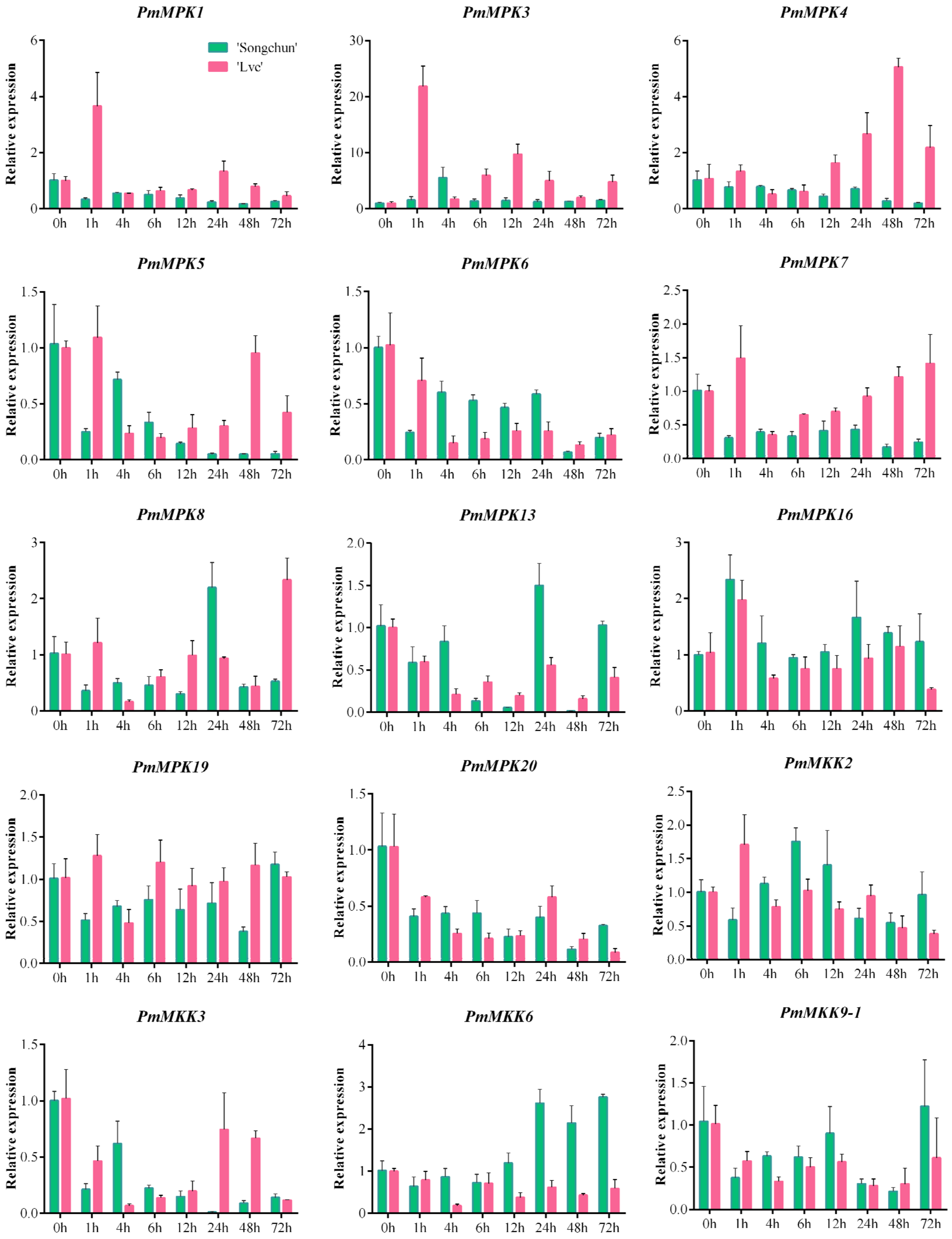

Figure S10 Expression patterns of 11 *PmMPK* and 4 *PmMKK* genes under low temperature treatments (*PmPP2A* as an internal reference gene)
