# Supplementary material for: Genome-Wide Identification of the MAPK and MAPKK Gene Families in Response to Cold Stress in Prunus mume
Source: Int J Mol Sci. 2023 May 16;24(10):8829. doi: 10.3390/ijms24108829 (PMC10218611; doi:10.3390/ijms24108829)
Supplement: Supplementary file 1 [file ijms-24-08829-s001.zip › ijms-2209574-supplementary/Supplement File S1 Protein sequences of PmMAPK and PmvMAPK.pdf]

Protein sequences of *PmMPK* and *PmvMPK*

>Pm000736(PmMPK13)
MESEDKVKREGLPLYGGKYIQYNILGNLFEVSAKYVPPIHPVGRGAYGIV
CCATNSETKEEVAIKKIGNAFDNRIDAKRTLREIKLLCHMDHDNIVKIKD
IIRPAEREKFNDVYIVYELMDTDLNQIIHSSQALTDDHCQYFLYQLLRGL
KYIHSANVLHRDLKPSNLLLNANCDLKICDFGLARTTSETDFMTEYVVTR
WYRAPELLLNCSEYTAAIDIWSVGCIFMEIIRREPLFPGKDYVQQLSLIT
ELLGSPDDSDLGFLRSDNARKYVKQLPHVPKQPFVQKFPNVSPLAIDLAE
KMLVFDPSKRITVEEALNHPFLSSLHEINEEPVCPSPFVFDFEQATLDEE
DIKELVWRESLHFNLDDMLG
>Pm000966(PmMPK3)
MADVPPSSGDFPAVPSHGGQYIQYNIFGNLFEITNKYRPPIMPIGRGAYG
IVCSVLNSETKEMVAMKKIANAFDNHMDAKRTLREIKLLRHLDHENVVAI
RDVIPPPLRREFSDVYIATELMDTDLHQIIRSNQGLSEEHCQYFLYQILR
GLKYIHSANVIHRDMKPSNLLLNANCDLKICDFGLARPTAENELLTEYVV
TRWYRAPELLLNSSDYTAAIDVWSVGCIFMELMNRKPLFPGKDHVHQMRL
LTELLGTPTESDLGFVRNEDARRYIRQLPPHPRQPLARLFPHVNPLAIDL
IDRMLTFDPTKRITVEEALAHPYLERLHDVADEPICNEPFSFDFEQQPLG
EEQMKDMIYREAIALNPEYA
>Pm005869(PmMPK1)
MATQVEPPNGIRSQGKHYYSMWQTLFEIDTKYVPIKPIGRGAYGIVCSSV
NRETNEKVAIKKINNAFENRIDALRTLRELKLLQHLRHENVIALKDVMVP
VQRKSFKDVYLVYELMDTDLHQIIKSSQPLSNDHCQYFLFQLLRGLKYLH
SANILHRDLKPGNLLINANCELKICDFGLARTSTGKGQFMTEYVVTRWYR
APELLLCCDNYGTSIDVWSVGCIFAELLGRKPIFPGTECLNQLKLIINIL
GSQREEDLQFIDNHKAKKYIRSLPYSLGTPFSRLYPDAHPLAIDLLQKML
VFDPSKRISVLEALQHPYMSALYDPSNNPPAEVPIDLDIDEDLGEETIRE
MMWKEMLHYHPEAAAGNAELFS
>Pm008036(PmMPK16)
MQSSADVDFFTEYGEGSRYKIEEVIGKGSYGVVCSAFDTHTGEKVAIKKI
NDIFEHVSDATRILREIKLLRLLRHPDIVEIKHILLPPSRREFKDIYVVF
ELMESDLHQVIKANDDLTPEHYQFFLYQLLRGMKYIHTANVFHRDLKPKN
ILANADCKLKICDFGLARVAFNDTPTAIFWTDYVATRWYRAPELCGSFFS
KYTPAIDVWSIGCIFAELLTGKPLFPGKNVVHQLDLMTDLLGTPSAEAIA
RVRNEKARRYLSSMRKKKQIPFSHKFPNADPLALRLLEKMLAFEPKDRPT
AEEALADPYFKGLAKVEREPSAQPVTKMEFEFERRRITKEDVRELIYRET
LEYHPKMLKEYLEGSEPTGFMYPSAVDHFKKQFAYLEEHYGNGATVVPPE
RTHASLPRACVLYSDNTVQHSTEVADDLSKCCIKEIEKPQIDRSSGIPTT
RLPVPQTIQVLDAGAARPGKVVGSVLRFNNCGAAAAAEALEQRRMVRNPT
AQPQYTATSTGSYPRRNPPCKNERGDCEGVEGSNGLQPKPQYMPRKVAAA
QGGSGQWY
>Pm011269(PmMPK19)

MDFFTEYGDANRYKILEVIGKGSYGVVCAAIDTHTGEKVAIKKIHDIFEH
ISDAIRILREVKLLRLLRHPDIVEIKRIMLPPSKREFKDIYVVFELMESD
LHQVIKANDDLTREHHQFFLYQMLRALKYMHTANVYHRDLKPKNILANAN
CKLKVCDFGLARVAFSDTPTTIFWTDYVATRWYRAPELCGSFFSKYTPAI
DIWSIGCIFAEVLTGKPLFPGKSVIHQLDLITDLLGTPKLETVSGVRNEK
ARKYLTEMRKKPLVPFTQKFPKADPLALRLLQRLLAFDPKDRPTAEEALA
DPYFKGLAKVEREHSCQPISKLEFEFERRRVTKEDIRELIYREILEYHPQ
LLKDYLNGTEGTSFLYPSAIGQFRKQFAYLEENSGKSGPVIPLARKHVSL
PRSTVHSSTIPPNAQPNLISYENRQAEEASSNFRVTDAISGNASKVLRPP
PRVPTAKPGRVVGPVLPYENGRNIKETYDPRTFYRNAVLPPQSVSPHCFF
RTHTANQDKSGLEMRRDASHAKLQPQPEQRNLAAKPAPGMAIEVNTNPYY
PPPTKADHLHVIDSKLLQAQSQFGPVGAAAVAVAAHRNTGTVQYGLS

>Pm014593(PmMPK20)

MDFFSDYGDANRYKIQEVIGKGSYGVVCSAIDTHTGEKVAIKKIHDIFEH
ISDAARILREIKLLRLLRHPDIVEIKHIMLPPSRRDFKDIYVVFELMESD
LHQVIKANDDLTREHYQFFLYQLLRALKYIHTANVYHRDLKPKNILANAN
CKLKICDFGLARVAFNDTPTTIFWTDYVATRWYRAPELCGSFFSKYTPAI
DIWSIGCIFAEVLTGKPLFPGKNVVHQLDLMTDLLGTPSLDTISRVRNEK
ARRYLTSMRKKQPVSLAQKFPNADPLALRLLERLLAFDPKDRPTAEEALA
DPYFKGLSRIEREPSCQPITKMEFEFERRRVTKEDIRELIFREILEYHPQ
LLKDYINGTERTNFLYPSAVDQFRKQFAHLEENGGKSGPVIPLERKHVSL
PRSTIVHSNTVPPKEQQNYAFLKDQKNAEEAYKNSRDNEGIHVNLSRTMQ
TPQRISLAKPGRVVGPVAQYENGNMMKDTYDRRTLVRSSVLPPQAGPPAY
CYRKPGAGNQERSVVEVERDMSSQAKQAAQCGMAANVAPDVAISIDSNPF
FMTRVGVSKVEHDDRIAVDTNYLQTKAPYGGIGAAAATAAAHRKVGTVQF
GMQRMY

>Pm018234(PmMPK4)

MESSSASAGDHNIKGVPAHGGRYVQYNVYGNFFEVSRKYVPPIRPVGRGA
YGIVCAAVNAETREEVAIKKIGNAFDNRIDAKRTLREIKLLRHMDHENVI
AIKDIIRPPQKENFNDVYIVYELMDTDLHQIIRSNQPLNDDHCRYFLYQL
LRGLKYVHSANVLHRDLKPSNLLMNANCDLKIGDFGLARTTSETDFMTEY
VVTRWYRAPELLLNCSEYTAAIDIWSVGCILGEIMTRRPLFPGKDYVHQL
RLITELLGSPDDSSLGFLRSDNARRYVRQLPQYPKQSFSVGFPNMSPSAV
DLLEKMLVFDPNRRISVDEALCHPYLAPLHDINEEPICPMPFNFDFEQPS
FTEENIKELIWRESVKFNPDPIH

>Pm023935(PmMPK5)

MAAKESSSASSTDAKIKRVLTHGGRYAQYNVFGNLFEVSSKYVPPIRPIG
RGAYGIVCAAVNADTHEEVAIKKIGNAFDNIIDAKRTLREIKLLCHMDHE
NVISIKDIVRPPKKETFNDVYIVYELMDTDLHQIIRSDQPLTDDHCQYFL
YQLLRGLKYVHSAHVLHRDLKPSNLFLNANCDLKIGDFGLARTTTETDFM
TEYVVTRWYRAPELLLNCSEYTAAIDIWSVGCILGEIMTREPLFPGKDYV
HQLRLITELIGSPDDASLGFLRSDNTRRYVKQLPQFQRQQFAARFPNMSP
GAVDLLEKMLVFDPNRRISVDEALSHPYLSSLHDNNDEPVCSRPFHFDFE
QPSCTEDHIKELIWRETVKFNPDPTH

>Pm025094(PmMPK8)
MALIWDRKREGCNSNTPQPQLEKIVELASSKRTHPPSMSMSMDENEMNKI
HACLRNTYMHLDTPDHPPYSQMIFKAINSLNYEEGGANKAAISAYIKSEF
HDLAWAHESLLSHHLGKLLERGELVTSPSTGDYQLPKPLLLAHEDLDPSK
HARGRGRRGRPPKPKRGNKENMTKRESLSLREQRKKQGINKYMGSGATLV
EGVRRWFQRRSSSTSTTINTNPNNNSKQPSNSNYSRNNFINDHNDAHVCV
SDLSAQSPTSHQREQKQDQEYQLQFEVEDDFDISGLKLISVPKRANFRAP
PMDSQKKGNLETEFFTEYGEASRYQVQEVIGKGSYGVVGSAIDTHTGEKV
AIKKINDVFEHVSDATRILREIKLLRLLRHPDIVEIKHIMLPPSRREFRD
IYVVFELMESDLHQVIKANDDLTPEHYQFFLYQLLRGLKYIHTANVFHRD
LKPKNILANADCKLKICDFGLARVSFNDAPSAIFWTDYVATRWYRAPELC
GSFFSKYTPAIDIWSIGCIFAEILTGKPLFPGKNVVHQLDLMTDLLGTPS
TESIARIRNEKARRYLSSMRKKQPVPFTHKFPHADPLALRLVEQLLAFDP
KDRPTAEEALADPYFHGLANVDREPSTQPISKLEFEFERRKLTKDDVREL
IYREILEYHPQMLQEYLRGGDQTSFMYPSGVDRFKRQFAHLEEHYGKGER
GTPPLQRQHASLPRERVCAPKDENSGQNNDVERTAASVASTLESPPGSQQ
PDGSVNADGQNGPSKTNHNARSLLKSASISASKCIGVRPKKDSEEEAIAE
VNDEAVDGLSQKVEALRA
>Pm026678(PmMPK7)
MATLVEPPDGIRQRGKHYYSMWQTLFEVDTKYVPIKPIGRGAYGIVCSSI
NRVTNEKVAIKKINNVFENRIDALRTLRELKLLRHIRHENVIALKDVMMP
IHRTSFKDVYFVYELMDTDLHQIIKSSQPLSSDHCKYFLFQLLRGLKYLH
SANILHRDLKPGNLLINANCDLKICDFGLARTSGGTGQFMTEYVVTRWYR
APELLLCCDNYGTSIDVWSVGCIFAEILGRKPIFPGTECLNQLKLIINVL
GSQHEPDLAFIDNPKARKYIKSLPYSRGTHFSRLYPQADPLAIDLLQRML
VFDPTKRISVTEALQHPYMSGLYDPRCNPPAQVPINLDIDENLAEPMIRE
MMWHEMLHYHPEAAFVNA
>Pm027774(PmMPK6)
MEARGSAAQSADTVMSEAAPPPAQADSTHPQQHPPPHPVMPGVESIPAT
LSHGGRFIQYNIFGNVFEVTAKYKPPIMPIGKGAYGIVCSALNSETNEHV
AIKKIANAFDNKIDAKRTLREIKLLRHMDHENVVAIRDIIPPPRRDQFND
VYIAYELMDTDLHQIIRSNQALSEEHCQYFLYQILRGLKYIHSANVLHRD
LKPSNLLLNANCDLKICDFGLARVTSETDFMTEYVVTRWYRAPELLLNSS
DYTAAIDVWSVGCIFMELMDRKPLFPGRDHVHQLRLLMELIGTPSETELG
FLNENAKRYIRQLPPHRRQSLTEKFPHVHPSAIDLVEKMLTFDPTKRITV
EDALAHPYLTSLHDISDEPVCMTPFSFDFEQHALTEEQMKELIYREALAF
NPEYQPQ
>PmuVar_Chr2_1968(PmvMPK1)
MATQVEPPNGIRSQGKHYYSMWQTLFEIDTKYVPIKPIGRGAYGIVCSSVNRETNEKVAI
KKINNAFENRIDALRTLRELKLLQHLRHENVIALKDVMVPVQRKSFKDVYLVYELMDTD
LHQIIKSSQPLSNDHCQYFLFQLLRGLKYLHSANILHRDLKPGNLLINANCELKICDFGLA
RTSTGKGQFMTEYVVTRWYRAPELLLCCDNYGTSIDVWSVGCIFAELLGRKPIFPGTECL
NQLKLIINILGSQREEDLQFIDNHKAKKYIRSLPYSLGTPFSRLYPDAHPLAIDLLQKML
VFDPSKRISVLEALQHPYMSALYDPSNNPPAEVPIDLDIDEDLGEETIREMMWKEMLHYH

PEAAAGNAELFS

>PmuVar_Chr2_2455(PmvMPK17)

MEAILRWFQGLSSSSSSSSADHRAISQSDVVQQPPSSASTDEQQEEELIITVELDMSGLK
PIKVPERTNHRLASMGHHKNMLDKEFFTEYGEASQYEIQEVIGKGSYGVVASAVDTHTGE
KVAIKKINDVFEHVSDATRILREIKLLRVLHHPDIVEIKHIMLPPCRREFKDIYVVFELM
ESDLHQVIKANDDLSPEHYQFFLYQLLRALKYIHTANVFHRDLKPKNILANSDCKLKICD
FGLARAAFSDAPSTIFWTDYVATRWYRAPELCGSFFSKYTPAIDIWSIGCIFAEMLTGKP
LFPGKNVVHQLDLITDLLGTPPAESISRIRNEKARRYLSSMKKKKSVPLSQKIPNADPLA
LRLLERLLAFDPRDRLSAVEALADPYFHGLANVNQEPSKQPISKLEFEFERSKLTKDDVR
ELIYREILEYHPKMLQDYLQGSDNIGFMYPSGVDRFRRQFAHLEAHYSKGERSTALQRKH
ASLPRERVCLSQDEAAEQNGNAKKSAAASVGRAAIHSPPRSKGFEVAESVCENGSTIPNG
LSKPNYSPNSLLKSASISASKCVAANRQYCEEDRTAERNDEMINVVA

>PmuVar_Chr2_5103(PmvMPK16)

MQSSADVDFFTEYGEGSRYKIEEVIGKGSYGVVCSAFDTHTGEKVAIKKINDIFEHVSDA
TRILREIKLLRLLRHPDIVEIKHILLPPSRREFKDIYVVFELMESDLHQVIKANDDLTPE
HYQFFLYQLLRGMKYIHTANVFHRDLKPKNILANADCKLKICDFGLARVAFNDTPTAIFW
TDYVATRWYRAPELCGSFFSKYTPAIDVWSIGCIFAELLTGKPLFPGKNVVHQLDLMTDL
LGTPSAEAIARVRNEKARRYLSSMRKKKQIPFSHKFPNADPLALRLLEKMLAFEPKDRPT
AEEALADPYFKGLAKVEREPSAQPVTKMEFEFERRRITKEDVRELIYRETLEYHPKMLKE
YLEGSEPTGFMYPSAVDHFKKQFAYLEEHYGNGATVVPPERTHASLPRACVLYSDNTVQH
STEVADDLSKCCIKEIEKPQIDRSSGIPTTRLPVPQTIQGAARPGKVVGSVLRFNNCGAA
AAAEALEQRRMVRNPTAQPQYTATSTGSYPRRNPPCKNERGDCEGVEGSNGLQPKPQYM
PRKVAAAQGGSGQWY

>PmuVar_Chr1_3483(PmvMPK13)

MESEDKVKREGLPLYGGKYIQYNILGNLFEVSAKYVPPIHPVGRGAYGIVCCATNSETKE
EVAIKKIGNAFDNRIDAKRTLREIKLLCHMDHDNIVKIKDIIRPAEREKFNDVYIVYELM
DTDLNQIIHSSQALTDDHCQYFLYQLLRGLKYIHSANVLHRDLKPSNLLLNANCDLKICD
FGLARTTSETDFMTEYVVTRWYRAPELLLNCSEYTAAIDIWSVGCIFMEIIRREPLFPGK
DYVQQLSLITELLGSPDDSDLGFLRSDNARKYVKQLPHVPKQPFVQKFPNVSPLAIDLAE
KMLVFDPSKRITVEEALNHPFLSSLHEINEEPVCPSPFVFDFEQATLDEEDIKELVWRES
LHFNLDDMLG

>PmuVar_Chr1_3496(PmvMPK3)

MADVPPSSGDFPAVPSHGGQYIQYNIFGNLFEITNKYRPPIMPIGRGAYGIVCSVLNSET
KEMVAMKKIANAFDNHMDAKRTLREIKLLRHLDHENVVAIRDVIPPPLRREFSDVYIATE
LMDTDLHQIIRSNQGLSEEHCQYFLYQILRGLKYIHSANVIHRDMKPSNLLLNANCDLKI
CDFGLARPTAENELLTEYVVTRWYRAPELLLNSSDYTAAIDVWSVGCIFMELMNRKPLFP
GKDHVHQMRLLTELLGTPTESDLGFVRNEDARRYIRQLPPHPRQPLARLFPHVNPLAIDL
IDRMLTFDPTKRITVEEALAHPYLERLHDVADEPICNEPFSFDFEQQPLGEEQMKDMIYR
EAIALNPEYA

>PmuVar_Chr5_2162(PmvMPK4)

MESSSASAGDHNIKGVPAHGGRYVQYNVYGNFFEVSRKYVPPIRPVGRGAYGIVCAAVN
AETREEVAIKKIGNAFDNRIDAKRTLREIKLLRHMDHENVIAIKDIIRPPQKENFNDVYIV
YELMDTDLHQIIRSNQPLNDDHCRYFLYQLLRGLKYVHSANVLHRDLKPSNLLMNANCD
LKIGDFGLARTTSETDFMTEYVVTRWYRAPELLLNCSEYTAAIDIWSVGCILGEIMTRRPL

FPGKDYVHQLRLITEAWFLNLACSPDDSSLGFLRSDNARRYVRQLPQYPKQSFSVGFPNM
SPSAVDLLEKMLVFDPNRRITVDEALCHPYLAPLHDINEEPICPMPFNFDFEQPSFTEEN
IKELIWRESVKFNPDPIH
>PmuVar_Chr3_1670(PmvMPK19)
MHADHLKKDLKDMDFFTEYGDANRYKILEVIGKGSYGVVCAAIDTHTGEKVAIKKIHDIF
EHISDAIRILREVKLLRLLRHPDIVEIKRIMLPPSKREFKDIYVVFELMESDLHQVIKAN
DDLTREHHQFFLYQMLRALKYMHTANVYHRDLKPKNILANANCKLKVCDFGLARVAFSD
TPTTIFWTDYVATRWYRAPELCGSFFSKYTPAIDIWSIGCIFAEVLTGKPLFPGKSVIHQL
DLITDLLGTPKLETVSGVRNEKARKYLTEMRKKPLVPFTQKFPKADPLALRLLQRLLAFD
PKDRPTAEEALADPYFKGLAKVEREHSCQPISKLEFEFERRRVTKEDIRELIYREILEYH
PQLLKDYLNGTEGTSFLYPSAIGQFRKQFAYLEENSGKSGPVIPLARKHVSLPRSTVHSS
TIPPNAQPNLISYENRQAEEASSNFRVTDAISGNASKVLRPPPRVPTAKPGRVVGPVLPY
ENGRNIKETYDPRTFYRNAVLPPQSVSPHCFFRTHTANQDKSGLEMRRDASHAKLQPQPE
QRNLAAKPAPGMAIEVNTNPYYPPPTKADHLHVIDSKLLQAQSQFGPVGAAAVAVAAHR
NTGTVQYGLS
>PmuVar_Chr4_3258(PmvMPK20)
MQQNQTEMDFFSDYGDANRYKIQEVIGKGSYGVVCSAIDTHTGEKVAIKKIHDIFEHISD
AARILREIKLLRLLRHPDIVEIKHIMLPPSRRDFKDIYVVFELMESDLHQVIKANDDLTR
EHYQFFLYQLLRALKYIHTANVYHRDLKPKNILANANCKLKICDFGLARVAFNDTPTTIF
WTDYVATRWYRAPELCGSFFSKYTPAIDIWSIGCIFAEVLTGKPLFPGKNVVHQLDLMTD
LLGTPSLDTISRVRNEKARRYLTSMRKKQPVSLAQKFPNADPLALRLLERLLAFDPKDRP
TAEEALADPYFKGLSRIEREPSCQPITKMEFEFERRRVTKEDIRELIFREILEYHPQLLK
DYINGTERTNFLYPSAVDQFRKQFAHLEENGGKSGPVIPLERKHVSLPRSTIVHSNTVPP
KEQQNYAFLKDQKNAEEAYKNSRDNEGIHVNLSRTMQTPQRISLAKPGRVVGPVAQYEN
GNMMKDTYDRRTLVRSSVLPPQAGPPAYCYRKPGAGNQERSVVEVERDMSSQAKQAAQ
CGMAANVAPDVAISIDSNPFFMTRVGVSKVEHDDRIAVDTNYLQTKAPYGGIGAAAATAA
AHRKVGTVQFGMQRMY
>PmuVar_Chr8_0208(PmvMPK6)
MEARGSAAQSADTVMSEAAPPPAQADSTHPQQHPPPPHPVIPGVESIPATLSHGGRFIQY
NIFGNVFEVTAKYKPPIMPIGKGAYGIVCSALNSETNEHVAIKKIANAFDNKIDAKRTLR
EIKLLRHMDHENVVAIRDIIPPPRRDQFNDVYIAYELMDTDLHQIIRSNQALSEEHCQYF
LYQILRGLKYIHSANVLHRDLKPSNLLLNANCDLKICDFGLARVTSETDFMTEYVVTRWY
RAPELLLNSSDYTAAIDVWSVGCIFMELMDRKPLFPGRDHVHQLRLLMELIGTPSETELG
FLNENAKRYIRQLPPHRRQSLTEKFPHVHPSAIDLVEKMLTFDPTKRITVEDALAHPYLT
SLHDISDEPVCMTPFSFDFEQHALTEEQMKELIYREALAFNPEYQPQ
>PmuVar_Chr8_1256(PmvMPK7)
MSVSLSARPVSDVEISEEEGKTRVASLRKKAMNASARFRKSLSKRGRRSSKVLSVEIEDV
HDSVELQAVDSLRQALILEELLPSKHDDYHMMLRFLKARKFDIEKTKQMWSDMLEWRK
EFGADTIMEDFEFKEHSEVLQHYPQGHHGVDKDGQPVYIERIGQVDVTKLMQATTMDRY
VKYHVREFERTFAVKFPACSIAAKKHIDQSTTILDVQGVGLKNFNKAARDLIARLQKIDGD
NYPETLNRMFIINAGPGFRMLWNTVKSFIDPKTAAKINVLGNKYQSKLLEIIDASELPEFLG
GTCTCADQGGCMRSDKGPWKKPEIMQMVQNGDHKCSRKSGIQERDTAPTLADKRPFSPR
FTMEHIEYPGLSRFQEEASIAKNFHESYKCKEHVPMVGKTVDSSWQMAGSSNFALSKGSS
SKNIAGDCFSRDDACKVPDGFGSQILAAVMAFVMGIVTMVRLTRNMPKKLTDSTFYSSTV

CDGDTMIKTQGPSYPAISGTDLMSVMKRMAELEERMSMLSVKPATVPAEKDRMLKTALG
RVDALEQELMATKQALEESLTRQDELLGSLDKKKKKKKNKINKKMESKCEVELLRLFRI
WIIEFDEAQVGPHPPSRLKLAKATGLGQLPLYTWRAACLREEKRKKMATLVEPPDGIRQR
GKHYYSMWQTLFEVDTKYVPIKPIGRGAYGIVCSSINRVTNEKVAIKKINNVFENRIDALR
TLRELKLLRHIRHENVIALKDVMMPIHRTSFKDVYFVYELMDTDLHQIIKSSQPLSSDHCK
YFLFQLLRGLKYLHSANILHRDLKPGNLLINANCDLKICDFGLARTSGGTGQFMTEYVVT
RWYRAPELLLCCDNYGTSIDVWSVGCIFAEILGRKPIFPGTECLNQLKLIINVLGSQHEPD
LAFIDNPKARKYIKSLPYSRGTHFSRLYPQADPLAIDLLQRMLVFDPTKRISVTEALQHP
YMSGLYDPRCNPPAQVPINLDIDENLAEPMIREMMWHEMLHYHPEAAFVNA
>PmuVar_Chr7_0255(PmvMPK12)
MAAKESSSASSTDAKIKRVLTHGGRYAQYNVFGNLFEVSSKYVPPIRPIGRGACGIVCAA
VNADTHEEVAIKKIGNAFDNIIDAKRTLREIKLLRHMDHENVISIKDIVRPPKKETFNDV
YIVYELMDTDLHQIIRSDQPLTDDHCQYFLYQLLRGLKYVHSAHVLHRDLKPSNLFLNAN
CDLKIGDFGLARTTTETDFMTEYVVTRWYRAPELLLNCSEYTAAIDIWSVGCILGEIMTR
EPLFPGKDYVHQLRLITELIGSPDDASLGFLRSDNTRRYVKQLPQFRRQQFAARFPNMSP
GAVDLLEKMLVFDPNRRISVDEALSHPYLSSLHDNNDEPVCSRPFHFDFEQPSCTEDHIK
ELIWRETVKFNPDPTH
>PmuVar_Chr7_1503(PmvMPK8)
MALIWDRKREGCNSNTPQLEKIVELASSKRTHPPSMSMDENEMNRIHACLRNTYMHLHT
PDHPPYSQEGGANKAAISAYIKSEFHDLAWAHENLLSHHLGKLLERGELVTSPSTGDYQLP
KPLLLAHEDLDPSKHARGRGRRGRPPKPKRGNKENMTKRESLSLREQRKKQGINKWFGV
IAPPCKVMGLSHNISVVCVAVNHQRLKGAEYMGSGATLVEGVRRWFQRRSSSTSTTINTN
PNNNSKQPSNSNYSRNNFINDHNDAHVCVSDLSAQSPTSHQREQKQDQEYQLQFEVEDD
FDISGLKLISVPKRANFRAPPMDSQKKGNLETEFFTEYGEASRYQVQEVIGKGSYGVVGS
AIDTHTGEKVAIKKINDVFEHVSDATRILREIKLLRLLRHPDIVEIKHIMLPPSRREFRDIY
VVFELMESDLHQVIKANDDLTPEHYQFFLYQLLRGLKYIHTANVFHRDLKPKNILANADC
KLKICDFGLARVSFNDAPSAIFWTDYVATRWYRAPELCGSFFSKYTPAIDIWSIGCIFAE
ILTGKPLFPGKNVVHQLDLMTDLLGTPSTESIARIRNEKARRYLSSMRKKQPVPFTHKFP
HADPLALRLVEQLLAFDPKDRPTAEEALADPYFHGLANVDREPSTQPISKLEFEFERRKL
TKDDVRELIYREILEYHPQMLQEYLRGGDQTSFMYPSGVDRFKRQFAHLEEHYGKGERG
TPPLQRQHASLPRERVCAPKDENSGQNNDVERTAASVASTLESPPGSQQPDGSVNADGQN
GPSKTNHNARSLLKSASISASKCIGVRPKKDSEEEAIAEVNDEAVDGLSQKVEALHA

Protein sequences of *PmMKK* and *PmvMKK*

>Pm007435(PmMKK9-2)
MALIRQRRQLNLRLPLPESSECRPCFSVPLPPTATVTAAVTNNSSFGSLSAADLEKLQVL
GHGNSGTVYKVNHKRTSDTYALKLVHGHSNDPTVRRQLFREMEILRRTDSPHVVRCHAIF
EKPSGDIGILMEYMDSGTLETLLKTQGTFSEPNLARVARQVLNGLNYLHTNKIIHRDIKP
ANVLVNRKMEVKIADFGVSKILCLTSDAYNSYVGTCAYMSPERFDPDTYGGNYNGYAGD
IWSLGLTLMELYMGHFPLLPPGQKPDWATLMCAICFGEPPSLPKGVSEEFRSFMECCLQKE
SEKRWTAAQLLTHPFVSADPSISIS
>Pm008654(PmMKK9-3)
MALIRQRRQLNLRLPLPEPSECRPCFSVPLPPITAVTNNSSFGTMSAADLEKLQVLGHGN

SGTVYKVNHKRTSTTYALKLVHGDSNDPTVRRQLFREMEILRRTDSPHVVRCHAIFEKPS
GDIGILMEYMDSGTLETLLKAQGTFSEPNLAHVARQVLNGLNYLHINKIIHRDIKPANVL
VNSNMEVKIADFGVSKILCRTLDACNSYVGTCAYMSPERFDPDTYGGNYNGYAGDIWSL
GLTLMELYMGHFPLLPPGQRPDWATLMCAICFGERPSLPEGVSEEFRSFMECCLQKESEKR
WTAAQLLTHPFVSKDRSISVPDGLLDLYASYRL

>Pm015648(PmMKK3)

MAGLEELRKKLTPLFDAEKGFSTGSTLDLDPCDSYTLSDSGTVNLLSRSYGVYNINELGL
QKCTTLAVDDSSEKTYRCGSHEMRIFGAIGSGASSVVQRAIHIPTHRILALKKINIFEKE
KRQQLLTEIRTLCEAPCYQGLVEFHGAFYTPDSGQISIALEYMDGGSLADILRLRKRIPE
PLLSSMFQKLLHGLSYLHGVRHLVHRDIKPANLLVNLKGEPKITDFGISAGLENSMAMCA
TFVGTVTYMSPERIRNENYSYPADIWSLGLALFECGTGEFPYTANEGPVNLMLQILDDPS
PTPPKHKFSPEFCSFIEACLQKDADARPTAEQLLSHPFITKYEDSQVDLAVFVRSVFDPT
QRMKDLADMLTIHFYLLFDGPDELWQHTRTLYSEDSVFSFSGKQLVGPNDIFASLSSIRR
TLAGDWPPERLVHVVEKLQCRAYGQDGVAIRVSGSFIVGNQFLICGDGVQVEGLPSIKDL
SIDISSKRMGTFQEQFIMEPSNKIGCYSISKQELYIMQ

>Pm023176(PmMKK10)

MTLVRERRHQQALRLSPPPPDVTPVAADHFRQQRTTTPTSTPDSPANVENLSDLEKLEVL
GHGNGGTVYKVCHKKTSTIYALKVLRFDHNGTGILQQAVREAEILKLVDSPYVIRCHGVF
DNNGFMSSTSDHNDGGGDLCFVMEYMEGGSLLDVLRARKRLPEHVISSVAKCVLQGLRY
LHAMQIVHRDIKPSNLLINGRAQVKIADFGVSHVVAGAREACDSYMGTYAYMSPERFDPE
RWGGDNADGFAGDVWSLGLVALQCHMGRFPLITPEQKPDWATLMCVICFGEGVEMPETA
SPEFQNFIWRCVEKDWRKRATVDELLDHPFVNKTCCSSTEDLVDYV

>Pm025044(PmMKK9-1)

MALIRERRQLNLRLPLPEPSECRPRFALPLPPTAAATTALTNNSSSAAISAADLDTLQVL
GHGNGGTVYKVRHKRTSTTYALKLVYGDSDPTVRRQIFREMEILRRTDSPYVVRCHAIFE
KPSGDIGIVMEYMDSGTLETLLKAHGTFSEPKLAHVARQVLNGLNYLHTHKIIHRDIKPT
NLLVNSNMEVKIADFGVSKIMCRTLDACNSYVGTCAYMSPERFDPDTYGGNYNGYASDI
WSLGLTLMELYMGHFPFLPPGQRPDWATLMCAICFGEPPSLPDGVSEEFRSFMECCLQKES
GKRWTAAQLLTHPFVCKDPSISVF

>Pm027015(PmMKK2)

MKKGSLGPNLKLTLPPPDEVSISKFLTKSGTFMDGDLLVNREGVRIVSHREVEVPPPIQP
SDNQMILADLYVIKVIGKGNGGVVQLVQHKWTGQFFALKVIQMNIEESIRKQIAQELKIN
QSSQCPYIVVCYQSFYDNGTISIILEYMDGGSLADLLKKVLQGLLYLHHEKHIIHRDFKP
SNLLINHQGEIKITDFGVSAIKASTSEQANTFVGTFNYMSPERIVGGNYSYKSDIWSLGL
VLLECATGQFPYLPPDQAQVWDSFFELMSAIVEQPPPCAPSDQFSHEFCSFISACVQKDP
KDRSSAQDLLTHPFINMYDDLNIDLASYFSEAGSPLATF

>Pm027289(PmMKK6)

MKTKTPLNLKQLELSVPAQEANIKSFLTASGTFHDGDLRLNQKGLRLISEEKEAQRNVPV
NVFMTSDSKELNFEISLEDLETIKVIGKGSGGVVQLVRHKWVGNLFALKVIQMNIQEEIR
KQIVQELKINQAAQCPHVVVCHHSFYHNGAISLVLEYMDRGSLADVIRQVKTILEPYLAV
VCKQVLQGLVYLHNERHVIHRDIKPSNLLVNHEGEVKITDFGVSASLASSMGQRDTFVGT
YNYMSPERISGSTYDYSSDIWSLGLVVLECAIGRFPYMQSEDQQSWPSFYELLEAIVESP
PPSAPSDQFSPEFCSFVSACIQKNPQDRSSSLDLLGHPFIKKFEDKDIDLGILVGSLEPP
VNFPR

>PmuVar_Chr2_4435(PmvMKK9-1)
MALIRQRRQLNLRLPLPEPSECRPCFSVPLPPITAVTNNSSFGTMSAADLEKLQVLGHGN
SGTVYKVNHKRTSTTYALKLVHGDSNDPTVRRQLFREMEILRRTDSPHVVRCHAIFEQPS
GDIGILMEYMDSGTLETLLKAQGTFSEPNLAHVARQVLNGLNYLHINKIIHRDIKPANVL
VNSNMEVKIADFGVSKILCRTLDACNSYVGTCAYMSPERFDPDTYGGNYNGYAGDIWSL
GLTLMELYMGHFPLLPPGQRPDWATLMCAICFGERPSLPDGVSEEFRSFMECCLQKESEKR
WTAAQLLTHPFVSKDRSISVS
>PmuVar_Chr2_5667(PmvMKK9-3)
MALIRQRRQLNLRLPLPESSECCPCFSVPLPPTATVTAAVTNNSSFGSISAADLEKLQVL
GHGNSGTVYKVNHKRTSDTYALKLVHGHSNDPTVRRQLFREMEILRRTDSPHVVRCHAIF
EKPSGDIGILMEYMDSGTLETLLKAQGTFSEPNLAHVARQVLNGLNYLHTNKIIHRDIKP
ANVLVNRKMEVKIADFGVSKILCLTSDACNSYVGTCAYMSPERFDPDTYGGNYNGYAGD
IWSLGLTLMELYMGHFPLLPPGQKPDWATLMCAICFGEPPSLPKGVSEEFRSFMECCLQKE
SEKRWTAAQLLTHPFVSTDPSISIS
>PmuVar_Chr4_2126(PmvMKK3)
MAGLEELRKKLTPLFDAEKGFSTGSTLDLDPCDSYTLSDSGTVNLLSRSYGVYNINELGL
QKCTTLAVDDSSEKTYRCGSHEMRIFGAIGSGASSVVQRAIHIPTHRILALKKINIFEKE
KRQQLLTEIRTLCEAPCYQGLVEFHGAFYTPDSGQISIALEYMDGGSLADILRLRKRIPE
PLLSSMFQKLLHGLSYLHGVRHLVHRDIKPANLLVNLKGEPKITDFGISAGLENSMAMCA
TFVGTVTYMSPERIRNENYSYPADIWSLGLALFECGTGEFPYTANEGPVNLMLQILDDPS
PTPPKHKFSPEFCSFIEACLQKDADARPTAEQLLSHPFITKYEDSQVDLAVFVRSVFDPT
QRMKDLADMLTIHYYLLFDGPDELWQHTRTLYSEDSVFSFSGKQLVGPNDIFASLSSIRR
TLAGDWPPERLVHVVEKLQCRAHGQDGVAIRVSGSFIVGNQFLICGDGVQVEGLPSIKDL
SIDISSKRMGTFQEQFIMEPSNIIGCYSISKQELYIMQ
>PmuVar_Chr8_0700(PmvMKK6)
MKTKTPLNLKQLELSVPAQEANIKSFLTASGTFHDGDLRLNQKGLRLISEEKEAQTSDSK
ELNFEISLEDLETIKVIGKGSGGVVQLVRHKWVGNLFALKVIQMNIQEEIRKQIVQELKI
NQAAQCPHVVVCHHSFYHNGAISLVLEYMDRGSLADVIRQVKTILEPYLAVVCKQVLQG
LVYLHNERHVIHRDIKPSNLLVNHEGEVKITDFGVSASLASSMGQRDTFVGTYNYMSPERI
SGSTYDYSSDIWSLGLVVLECAIGRFPYMQSEDQQSWPSFYELLEAIVESPPPSAPSDQF
SPEFCSFVSACIQKNPQDRSSSLDLLGHPFIKKFEDKDIDLGILVGSLEPPVNFPR
>PmuVar_Chr8_0833(PmvMKK2)
MKKGSLGPNLKLTLPPPDEVSISKFLTKSGTFMDGDLLVNREGVRIVSHREVEVPPPIQP
SDNQMILADLYVIKVIGKGNGGVVQLVQHKWTGQFFALKVIQMNIEESIRKQIAQELKIN
QSSQCPYIVVCYQSFYDNGTISIILEYMDGGSLADLLKKVKTIPEPYLASICKQVLQGLL
YLHHEKHIIHRDFKPSNLLINHQGEIKITDFGVSAIKASTSEQANTFVGTFNYMSPERIV
GGNYSYKSDIWSLGLVLLECATGQFPYLPPDQAQVWDSFFELMSAIVEQPPPCAPSDQFS
QEFCSFISACVQKDPKDRSSAQDLLTHPFINMYNDLNIDLASYFSEAGSPLATF
>PmuVar_Chr7_1550(PmvMKK9-2)
MALIRERRQLNLRLPLPEPSECRPRFALPLPPTAAATTALTNNSSSAAISAADLDTLQVL
GHGNGGTVYKVRHKRTSTTYALKLVYGDSDPTVRRQIFREMEILRRTDSPYVVRCHAIFE
KPSGDIGIVMEYMDSGTLETLLKAHGTFSEPKLAHVARQVLNGLNYLHTHKIIHRDIKPT
NLLVNSNMEVKIADFGVSKIMCRTLDACNSYVGTCAYMSPERFDPDTYGGNYNGYASDI
WSLGLTLMELYMGHFPFLPPGQRPDWATLMCAICFGEPPSLPDGVSEEFRSFMECCLQKES

GKRWTAAQLLTHPFVCKDPSISVF

>PmuVar_Chr7_1991(PmvMKK10)

MTLVRERRHQQALRLSPPPPDVTPVAADHFRQQRTTTPTSTPDSPANVENLSDLEKLEVL
GHGNGGTVYKVCHKKTSTIYALKVLRFDHNGTSILQQAVREAEILKLVDSPYVIRCHGVF
DNNGFMSSTSDHNDGGGDLCFVMEYMEGGSLLDVLRARKRLPEHVISSVAKCVLQGLH
YLHAMQIVHRDIKPSNLLINGRAQVKIADFGVSHVVAGAREACDSYMGTYAYMSPERFDP
ERWGGDNADGFAGDVWSLGLVALQCHMGRFPLITPEQKPDWATLMCVICFGEGVEMPET
ASPEFQSFIWRCVEKDWRKRATVDELLDHPFVNKTCCSSTEDLVDYV
